# Telemedicine for Follow-up Management of Patients After Liver Transplantation: Cohort Study

**DOI:** 10.2196/27175

**Published:** 2021-05-17

**Authors:** Min Tian, Bo Wang, Zhao Xue, Dinghui Dong, Xuemin Liu, Rongqian Wu, Liang Yu, Junxi Xiang, Xiaogang Zhang, Xufeng Zhang, Yi Lv

**Affiliations:** 1 Department of Hepatobiliary Surgery The First Affiliated Hospital of Xi’an Jiaotong University Xi’an China; 2 National Local Joint Engineering Research Center for Precision Surgery & Regenerative Medicine The First Affiliated Hospital of Xi’an Jiaotong University Xi’an China

**Keywords:** liver transplantation, optimal clinical outcomes, telemedicine, telemedical follow-up management system, telerobotics, rapid recovery, health-related quality of life, remote health monitoring, telehealth, cohort, mortality, morbidity

## Abstract

**Background:**

Technical capabilities for performing liver transplantation have developed rapidly; however, the lack of available livers has prompted the utilization of edge donor grafts, including those donated after circulatory death, older donors, and hepatic steatosis, thereby rendering it difficult to define optimal clinical outcomes.

**Objective:**

We aimed to investigate the efficacy of telemedicine for follow-up management after liver transplantation.

**Methods:**

To determine the efficacy of telemedicine for follow-up after liver transplantation, we performed a clinical observation cohort study to evaluate the rate of recovery, readmission rate within 30 days after discharge, mortality, and morbidity. Patients (n=110) who underwent liver transplantation (with livers from organ donation after citizen's death) were randomly assigned to receive either telemedicine-based follow-up management for 2 weeks in addition to the usual care or usual care follow-up only. Patients in the telemedicine group were given a robot free-of-charge for 2 weeks of follow-up. Using the robot, patients interacted daily, for approximately 20 minutes, with transplant specialists who assessed respiratory rate, electrocardiogram, blood pressure, oxygen saturation, and blood glucose level; asked patients about immunosuppressant medication use, diet, sleep, gastrointestinal function, exercise, and T-tube drainage; and recommended rehabilitation exercises.

**Results:**

No differences were detected between patients in the telemedicine group (n=52) and those in the usual care group (n=50) regarding age (*P*=.17), the model for end-stage liver disease score (MELD, *P*=.14), operation time (*P*=.51), blood loss (*P*=.07), and transfusion volume (*P*=.13). The length and expenses of the initial hospitalization (*P*=.03 and *P*=.049) were lower in the telemedicine group than they were in the usual care follow-up group. The number of patients with MELD score ≥30 before liver transplantation was greater in the usual care follow-up group than that in the telemedicine group. Furthermore, the readmission rate within 30 days after discharge was markedly lower in the telemedicine group than in the usual care follow-up group (*P*=.02). The postoperative survival rates at 12 months in the telemedicine group and the usual care follow-up group were 94.2% and 90.0% (*P*=.65), respectively. Warning signs of complications were detected early and treated in time in the telemedicine group. Furthermore, no significant difference was detected in the long-term visit cumulative survival rate between the two groups (*P*=.50).

**Conclusions:**

Rapid recovery and markedly lower readmission rates within 30 days after discharge were evident for telemedicine follow-up management of patients post–liver transplantation, which might be due to high-efficiency in perioperative and follow-up management. Moreover, telemedicine follow-up management promotes the self-management and medication adherence, which improves patients’ health-related quality of life and facilitates achieving optimal clinical outcomes in post–liver transplantation.

## Introduction

In 1967, Thomas Starzl performed the first successful liver transplantation [1]. Nearly half a century later, it has become a widely accepted treatment for end-stage liver disease and selected liver malignancies. Improvements in multiple dimensions, including refinement of explanting and organ preservation techniques, surgical techniques, perioperative care, and the development of potent immunosuppressive drugs have improved the outcomes of liver transplantation with 1-year survival rates >85% [2,3] and the 5-year survival rate approaching 75% [4]. The success of liver transplantation has led to an expansion of indications [5-7]; however, the lack of availability of the critical organ has prompted the use of edge donor grafts [8], such as those donated after circulatory death, from older donors, and from with hepatic steatosis [9,10]. In the past 20 years, the capabilities for liver transplantation have made remarkable progress in China. The perioperative mortality rate has been reduced to <5%, and the postoperative survival rates at 1, 5, and 10 years have reached 90%, 80%, and 70%, respectively. In 2006, the liver transplantation team led by Shu-sen Zheng at the First Affiliated Hospital, School of Medicine, Zhejiang University proposed the Hangzhou criteria [11]. Comparison of the 1-, 3-, and 5-year survival rates between Milan criteria and Hangzhou criteria groups did not reveal any statistical differences [12]. The technical capabilities for liver transplantation in China, the postoperative graft survival rate, and recipient survival rate are on par with those of the global level [13].

The increasing complexities in the liver transplantation process make it difficult to determine optimal clinical outcomes. *Textbook outcome* is an emerging concept within multiple surgical domains that defines a standardized composite quality benchmark based on multiple endpoints perioperatively, representing the ideal textbook hospitalization [14]. Although the definition of textbook outcome varies, it frequently includes the evaluation of morbidity, mortality, length of stay, and hospital readmission. Moris et al [15] defined textbook outcome as a metric of an ideal outcome in liver transplantation. The textbook outcome for liver transplantation is based on the exclusion of the following parameters: mortality within 90 days, primary allograft nonfunction, early allograft dysfunction, rejection of the graft within 30 days, readmission with 30 days, readmission to the intensive care unit during hospitalization, hospital length of stay >75th percentile of all liver transplantation, red blood cell transfusion requirement >75th percentile for all liver transplantation complications (reintervention), and major intraoperative complications. We speculate that the achievement of textbook outcome in liver transplantation is a composite metric reflecting the quality of perioperative care and cost-effective practice. Therefore, the perioperative management and follow-up system in liver transplantation are under intensive focus.

Telemedicine is the dissemination of health services over long distances by health care providers using information and communication technology [16]. eHealth is an efficient and cost-eﬀective alternative to traditional health care that can be used to improve patients’ health-related quality of life and satisfaction [17]. Telemedicine is driven by rapid developments in medicine, information, and communication technology. It has been used for many diseases (chronic obstructive pulmonary disease, asthma, heart failure) because it facilitates real-time consultation between caregivers and patients to provide timely and improved personalized care. Telemedicine also facilitates diagnosis and treatment options when medical evacuation is impossible due to acute medical emergencies, mass casualty disasters, and public health measures (such as during COVID-19 pandemic restrictions) [18,19]. From a global health perspective, telemedicine increases the availability and quality of health care in remote areas and reduces medical inequalities between remote and urban areas [20-24]. Changes in the medical field have prompted concerns—how to achieve the optimal clinical outcome (ie, textbook outcome) in liver transplantation? What is required to establish a new model to meet the challenge of the new era?

The greatest strength of telemedicine is to provide face-to-face communication in over long distances for specialized health care services, thereby eliminating the need for both the physician and patient being in the same location. We aimed to investigate the efficacy of a telemedicine follow-up management intervention after liver transplantation on recovery, hospital readmission, mortality, and morbidity.

## Methods

### Study Design and Participants

We conducted a clinical observation study. Between January 1, 2015 and September 30, 2018, a total of 340 patients underwent orthotopic liver transplantation in the First Affiliated Hospital of Xi’an Jiao Tong University, Shaanxi, China. The livers were donated after citizen’s death. The patients were eligible for inclusion in the study if they fulfilled the discharge conditions for orthotopic liver transplantation (stable liver function and immunosuppressant blood concentration, improved diet and exercise), were willing to participate telemedicine-based follow-up management, and provided written informed consent. Patients were excluded from the study if they did not have a wireless network at home.

Patients who were enrolled in this study were randomly assigned after hospital discharge to either telemedicine-based follow-up management for 2 weeks in addition to the usual care or usual care follow-up only. All patients were followed up for 12 months, and long-term survival follow-up data were recorded until December 31, 2020.

This study was approved by the First Affiliated Hospital of Xi’an Jiao Tong University Ethics Committee (XJTU1AF2020LSK-171) and conducted in compliance with the Declaration of Helsinki and the International Code of Medical Ethics.

### Patients, Health Care Professionals, and Facilities Involved in the Telemedicine Follow-up Management System

**Figure 1:**
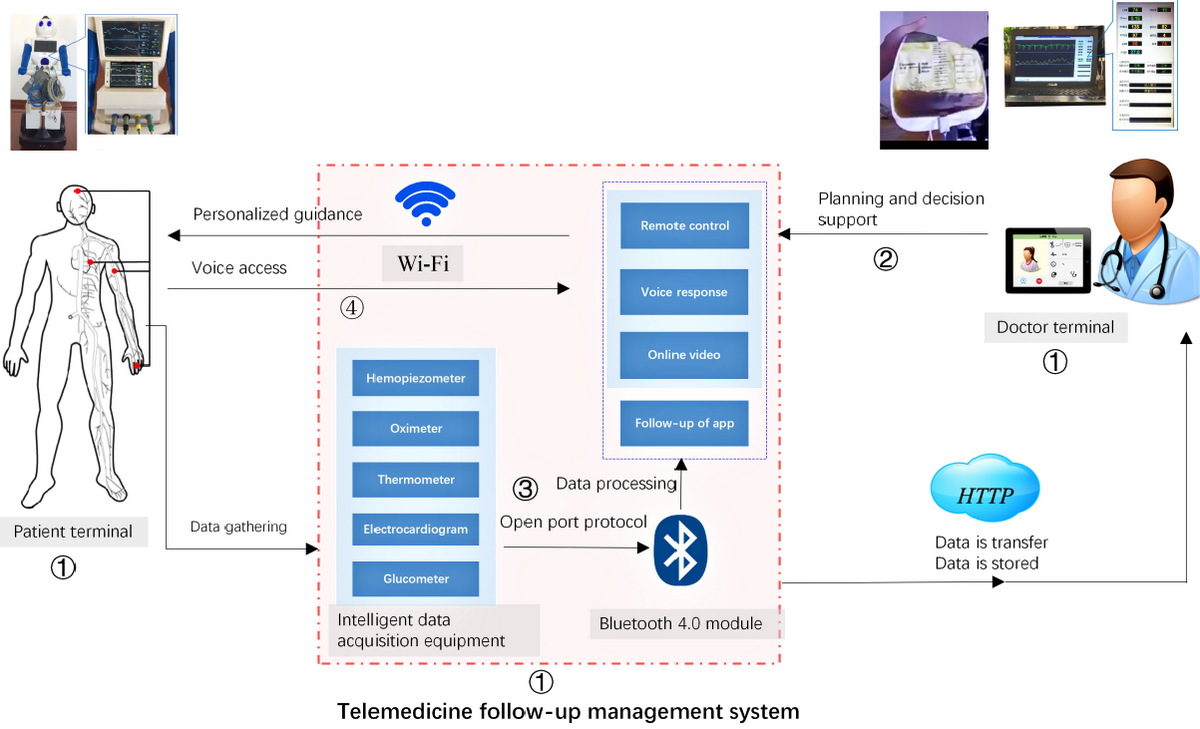
Schematic of the telemedicine follow-up management system. ① The telemedicine follow-up management system includes doctor-terminal, patient-terminal, and management platform. ② The transplant specialist remotely controlled the intelligent robot “face-to-face” communication with patients by a computer, mobile phone, and tablet from anywhere, such as monitoring vital signs and T tube drainage. ③ Based on Internet-based telecommunication systems, the physiological parameters, such as respiratory rate, ECG, blood pressure, oxygen saturations processed, blood sugar or authorized by transplant specialists with feedback to the patients, were telemonitored by wireless equipment. ④ The patients could communicate with the transplant specialists about the examination results through the telemedicine follow-up management system in real-time or using store-and-forward technology.

The telemedicine follow-up management system (Figure 1) included doctor terminal app, patient terminal app, and management platforms. The data acquisition equipment and intelligent service robot were utilized to acquire blood pressure, blood oxygen, temperature, and electrocardiography (ECG) data. The data transmission between the monitoring equipment and the robot used an Android Bluetooth interface. Internet-based telecommunication with health care professionals [25] used video or telephone links in real-time, and store-and-forward technology was applied [26]. The transplant specialist remotely controlled the intelligent robot face-to-face communication with liver transplantation recipients using a computer, mobile phone, or iPad. Patients’ physiological parameters, such as respiratory rate, ECG, blood pressure, oxygen saturation, blood glucose level, and feedback were telemonitored via the wireless equipment [27]. The rehabilitation programs were administered after liver transplantation with home-based video conference supervised exercise, and counseling by transplant professionals.

### User Training

Before initiating this study, we piloted the telemedicine follow-up management system in healthy volunteers to evaluate the feasibility of the system. Both remote transplant specialists and patients were trained to use this system. The average training time for patients was 1 hour. The acceptance of this model was based on the response to a yes or no questionnaire given to the specialists and patients, and a criterion was defined that acceptance should reach >95%.

### Telemedicine Follow-up Management Intervention

Patients in this group received telemedicine follow-up management in the first 2 weeks after hospital discharge. Patients were discharged, and the telemedicine follow-up robot was given to them to take home free-of-charge.

The transplant specialists called the patients to turn on the telemedicine follow-up management robot at a specific time every morning. The transplant specialist remotely controlled the intelligent robot via face-to-face communication with liver transplantation recipients using a computer, mobile phone, or iPad. The patient used the equipment of the telemedicine follow-up robot to capture their vital signs (respiratory rate, ECG, blood pressure, oxygen saturation) and blood glucose level.

While monitoring patients’ vital data, the transplant specialists inquired about the medication of the immunosuppressive agents after discharge, daily diet, sleep, relief of the bowels, exercise, and drainage of the T tube; provided guidance, and initiated rehabilitation programs for the patients. Each daily session lasted approximately 20 minutes.

The patient visited the outpatient service weekly during the 2 weeks for examination of immunosuppressant blood concentration and biochemical indexes (such as liver function) and for color doppler ultrasonography of the graft. The patients could communicate with the transplant specialists about examination results and drug adjustments through the telemedicine follow-up management system. After the end of the 2-week period, patients returned the telemedicine follow-up robot to the hospital and continued routine outpatient follow-up.

### Usual Care Follow-up

The patients in the usual care follow-up group attended outpatient follow-up visits each week in the first month after hospital discharge for examination of immunosuppressant blood concentration and biochemical indexes (such as liver function) and for color doppler ultrasonography of the graft. Outpatient follow-up visits occurred every 2 weeks after the first month, then every month in the first half-year, and thereafter, every 2 to 3 months.

### Statistical Analysis

Continuous variables are reported as mean and standard deviation. Categorical variables are presented as frequency and percentages and were compared using one-way analysis of variance. Survival was evaluated using Kaplan-Meier curves. A *P* value <.05 was considered statistically significant. All statistical analyses were performed using SPSS statistical software (version 20; IBM Corp).

## Results

### Participants

**Figure 2:**
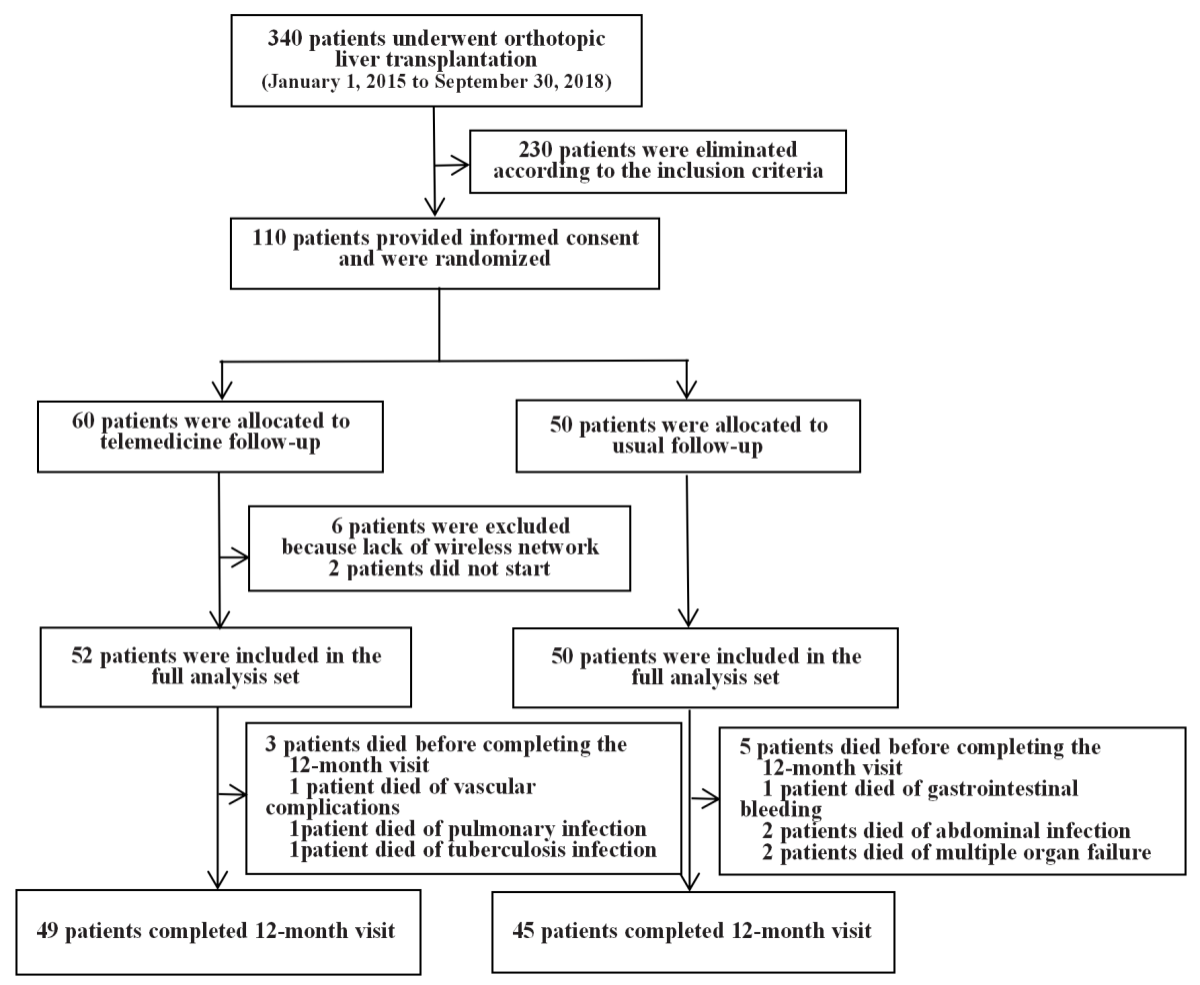
Procedures and participants in the telemedicine follow-up management clinical observation study.

A total of 340 patients underwent liver transplantation between January 1, 2015 and September 30, 2018; 110 patients were included in this study. A total of 60 patients were eligible for inclusion in the telemedicine group, but 6 patients were excluded from the study because they did not have a wireless network at home, 2 patients did not start the program because they could not use the telemedicine follow-up management system, and the other 52 patients were included in the full analysis set; 50 patients in the usual care follow-up group were included in the full analysis set. All patients were followed up for 12 months, and long-term follow-up data were recorded (Figure 2). 

### Baseline Characteristics

Patient characteristics are reported in Table 1. Of the 102 patients, the mean age was 46.65 (SD 9.66) years, and 72 (70.6%) patients were male. Of the 52 patients in the telemedicine group, the mean age was 45.35 (SD 10.44) years, and 40 (76.9%) patients were male. Of the 50 patients in the usual care follow-up group, the mean age was 48.00 (SD 8.68) years, and 32 (64.0%) patients were male. No significant differences were found for age (*P*=.17) and sex (*P*=.16) between the two groups. Malignant tumor disease before liver transplantation was observed in 20/52 (38.5%) patients in the telemedicine group and in 19/50 (38.0%) patients in the usual care follow-up group (*P*=.96). The model for end-stage liver disease (MELD) score before liver transplantation in the telemedicine group and the usual care follow-up group did not differ significantly (*P*=.14). In further analysis, 38 (73.1%) patients, 10 (19.2%) patients, and 4 (7.7%) patients in the telemedicine group and 31 (62.0%) patients, 9 (18.0%) patients, and 10 (20.0%) patients in usual care follow-up group had MELD scores <20, 20-30, and ≥30, respectively, before liver transplantation.

**Table 1:** Baseline characteristics.

			Total (N=102)		Telemedicine management intervention (n=52)		Usual care (n=50)		*P* value
Age (years), mean (SD)			46.65 (9.66)		45.35 (10.44)		48.00 (8.68)		.17
**Sex, n (%)**									.16
	Male	72 (70.6)		40 (76.9)		32 (64.0)			
	Female	30 (29.4)		12 (23.1)		18 (36.0)			
**Diagnosis, n (%)**									.96
	Malignant diseases	39 (38.2)		20 (38.5)		19 (38.0)			
	Benign disease	63 (61.8)		32 (61.5)		31 (62.0)			
MELD^a^ score, mean (SD)			18.03 (8.78)		16.77 (7.86)		19.34 (9.55)		.14
**MELD score, n (%)**									.13
	<20	69 (67.7)		38 (73.1)		31 (62.0)			
	20-30	19 (19.6)		10 (19.2)		9 (18.0)			
	≥30	14 (12.7)		4 (7.7)		10 (20.0)			
Donor age (years), mean (SD)			47.21 (14.66)		43.44 (14.51)		51.12 (13.91)		.008
**Donor age (years), n (%)**									.003
	<18	3 (2.9)		3 (5.8)		0 (0)			
	18-65	87 (85.3)		47 (90.4)		40 (80.0)			
	≥65	12 (11.8)		2 (3.8)		10 (20.0)			
Orthotopic liver transplantation operation time (hours), mean (SD)			6.33 (1.00)		6.27 (1.01)		6.40 (1.00)		.51
Blood loss (mL), mean (SD)			1462.26 (1280.54)		1234.62 (945.55)		1699.00 (1528.79)		.07
Transfusion volume (mL), mean (SD)			5948.92 (1733.48)		5694.17 (1457.13)		6213.84 (1960.49)		.13
Length of initial hospitalization (days), mean(SD)			17.69 (6.56)		16.31 (3.57)		19.12 (8.45)		.03
Expense of initial hospitalization (Yuan^b^), mean (SD)			395094 (66101.04)		382502.36 (35115.42)		408190.11 (85904.13)		.049
Readmission rate within 30 days after discharge, mean (SD)			0.16 (0.37)		0.08 (0.27)		0.24 (0.43)		.02
Survival rate (%) at 12-month visit, mean (SD)			94 (92.2)		49 (94.2)		45 (90.0)		.65

^a^MELD: Model for End-Stage Liver Disease

^b^An approximate exchange rate of 6.48 Yuan=US $1 was applicable at the time of publication.

Livers donation after citizen’s death are currently the primary source of donors in China [28]. The donor age in the telemedicine group was lower than that in the usual care follow-up group (*P*=.008). Further analysis revealed that 2/52 (3.85%) in the telemedicine group, while 10/50 (20%) patients in the usual care follow-up group were older adult (>65 years old) donors.

### Primary and Key Secondary Outcomes

**Figure 3:**
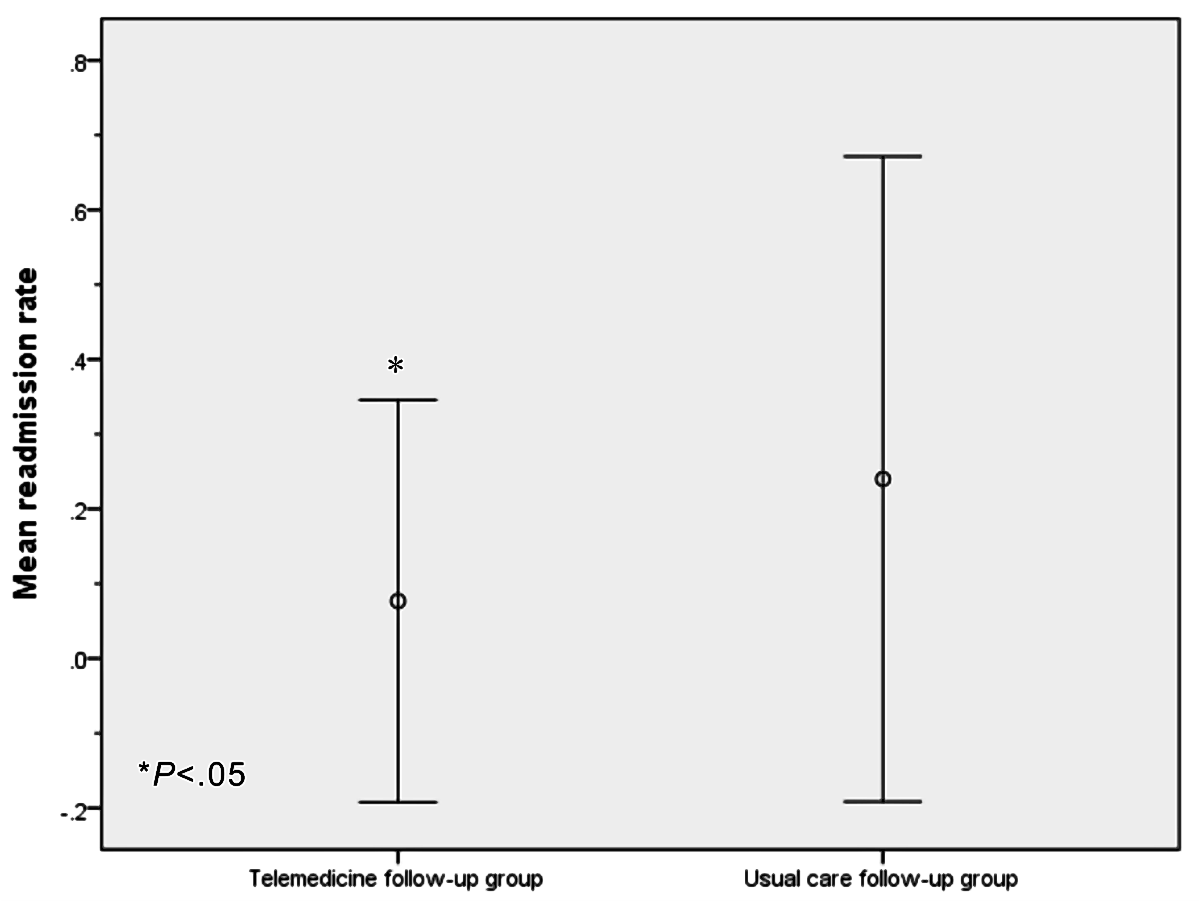
The mean readmission rate within 30 days after discharge in the two groups. Readmission rate within 30 days after discharge in the telemedicine follow-up group was markedly lower than that in the usual care follow-up group (telemedicine: mean 0.08, SD 0.27; usual care: mean 0.24, SD 0.43; *P*=.02).

No difference were found between the telemedicine and the usual care follow-up group with respect to operation time (*P*=.51), blood loss (*P*=.07), and intraoperative transfusion volume (*P*=.13); the operation quality parameters of liver transplantation in the two groups were similar. Nevertheless, statistically significant differences were found in the length of initial hospitalization (telemedicine: mean 16.31, SD 3.57; usual care: mean 19.12, SD 8.45; *P*=.03) and initial hospitalization expense (telemedicine: mean 382502.36 Yuan, SD 35115.42; usual care: mean 408190.11 Yuan SD 85904.13, an approximate exchange rate of 6.48 Yuan=US $1 was applicable at the time of publication; *P*=.049). The number of patients with MELD score ≥30 before liver transplantation was greater in the usual care follow-up group than that in telemedicine follow-up group. Furthermore, the readmission rate within 30 days after discharge was markedly lower in the telemedicine group than that in the usual care follow-up group (telemedicine: mean 0.08, SD 0.27; usual care: mean 0.24, SD 0.43; *P*=.02) (Figure 3).

In the telemedicine group, 3 patients died before the 12-month visit (vascular complications: n=1, pulmonary infection: n=1, and tuberculosis infection: n=1); the postoperative survival rate at 12 months was 94.2%. In the usual care follow-up group, 5 patients died (portal vein thrombosis that led to gastrointestinal bleeding: n=1, severe abdominal infection: n=2, multiple organ failure: n=2); the postoperative survival rate at 12 months was 90.0% (Figure 2). There was no significant difference in the 12-month cumulative survival rate between the two groups (*P*=.65).

### Major Complications After Liver Transplantation

Occurrences of significant complications, such as primary graft failure, primary graft dysfunction, acute rejection reaction, vascular complications, biliary complications, tumor recurrence, and severe infection, after liver transplantation of patients at the 12-month follow-up did not differ significantly between the two groups (Table 2).

One patient in the telemedicine group (male; 37 years old; acute-on-chronic liver failure, hepatitis B, and cirrhosis) underwent liver transplantation on August 12, 2016. He had severe postoperative complications, such as primary graft dysfunction. The patient was treated with methylprednisolone combined with multiple plasmapheresis, as well as anti-infection and liver protection. The patient recovered, was discharged after 39 days of hospitalization, and enrolled in the telemedicine group to gain guidance for postoperative rehabilitation and follow-up. At the end of the study, he was alive and healthy. 

Three (6.0%) patients in the follow-up group had portal vein thrombosis, and underwent interventional thrombolysis and portal vein stents immediately; however, these were not effective, and 1 patient died of gastrointestinal bleeding. Although portal vein thrombosis did not occur in any patients in the telemedicine group, 3 patients exhibited portal vein stenosis in the telemedicine group; thus, it was recommended by the transplant specialists of the telemedicine follow-up management that these patients be readmitted; they were readmitted for portal vein angiography and portal vein stent implantation and survived.

**Table 2:** Major complications after liver transplantation of patients at the 12-month follow-up visit in the two groups.

Groups	All (N=102), n	Telemedicine management intervention (n=52), n (%)	Usual care (n=50), n (%)	*P* value
Primary graft failure	0	0 (0)	0 (0)	N/A^a^
Primary graft dysfunction	1	1 (1.9)	0 (0)	.33
Acute rejection reaction	7	4 (7.7)	3 (6.0)	.74
Hepatic artery thrombosis	5	3 (5.8)	2 (4.0)	.68
Portal vein thrombosis	3	0 (0)	3 (6.0)	.07
Severe biliary complications	11	4 (7.7)	7 (14.0)	.31
Tumor recurrence	7	3 (5.8)	4 (8.0)	.66
Serious infection	4	2 (3.9)	2 (4.0)	.97

^a^N/A: not applicable.

The biliary complications were common complications of liver transplantation and required repeated endoscopic retrograde cholangiopancreatography procedures or preoperative biliary drainage: 4 (7.7%) patients in the telemedicine group and 7 (14.0%) patients in the usual care follow-up group with benign biliary stricture or bile leakage. We found that magnetic compression anastomosis was a minimally invasive method of performing choledochostomy for benign biliary stricture. One patient in the telemedicine group had benign biliary stricture, and hence, we attempted a variety of conventional treatments that failed, following which, the patient underwent preoperative biliary drainage before magnetic compression anastomosis. The device consisted of a parent and a daughter magnet. The daughter magnet was delivered via the preoperative biliary drainage route to the proximal end of the obstruction, and the parent magnet was delivered via endoscopic retrograde cholangiopancreatography to the distal end of the obstruction. After recanalization, the magnetic compression anastomosis device was removed, and biliary stenting was performed for at least 6 months with complete resolution of the condition [29,30]. Additionally, 2 patients with bile leakage detected at the telemedicine follow-up management were admitted immediately for endoscopic retrograde cholangiopancreatography and a biliary stent implanted under the guidance of transplant specialists.

### Long-term Survival Analysis

**Figure 4:**
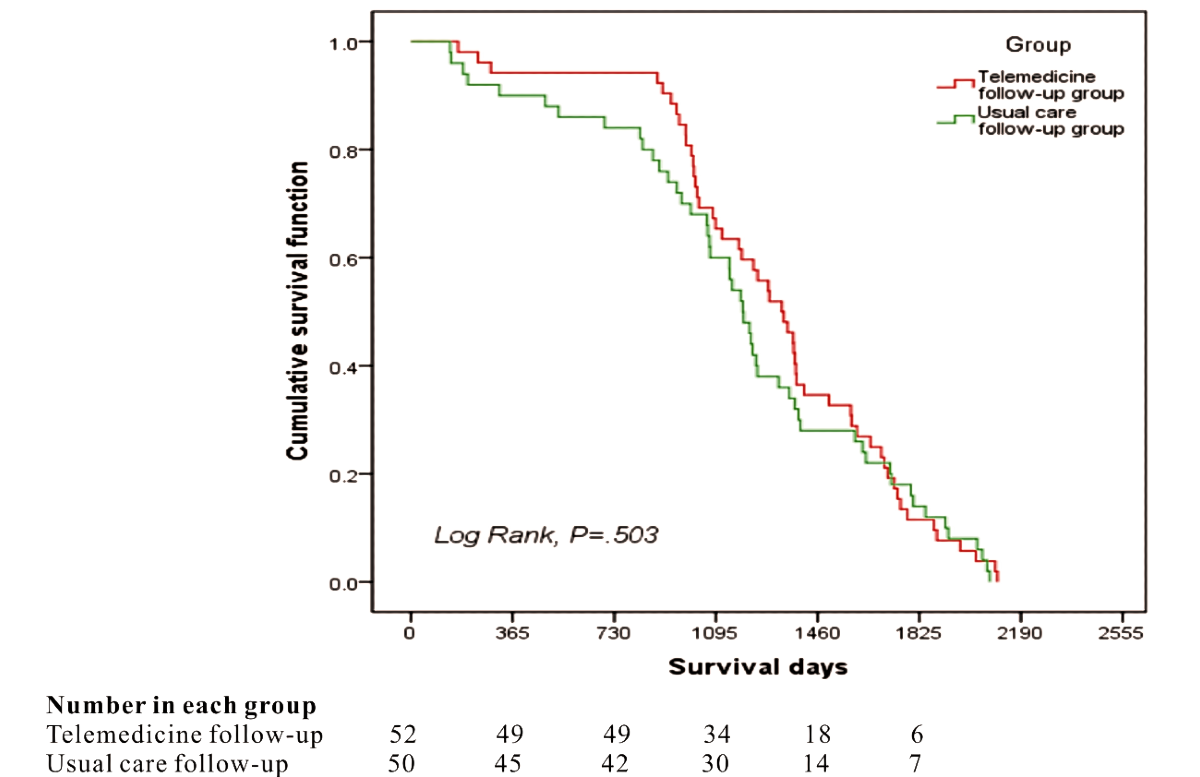
The cumulative survival curves for both groups in the long-term follow-up. No significant difference was detected in the cumulative survival rate between the two groups (*P*=.503).

Since the patients who encountered liver transplantation were followed up for life, the two groups of patients in this study were monitored continually. Long-term survival follow-up data have been recorded up until December 31, 2020. 4 patients have died in the telemedicine group, and 10 patients have died in the usual care follow-up group. The majority of these patients exhibited tumor recurrence and included other post–liver transplantation complications, such as lymphoma and cholestatic cirrhosis. None died in the perioperative period. The postoperative survival rates in the telemedicine group at 1, 2, and 3 years were 94.2%, 94.2%, and 65.4%, respectively. The postoperative survival rates in the usual care follow-up group at 1, 2, and 3 years were 90.0%, 84.0%, and 60.0%, respectively; however, no significant differences were detected between the cumulative survival curves of the two groups (*P*=.50) (Figure 4).

## Discussion

### Principal Findings

Rapid recovery and lower readmission rate within 30 days after discharge were evident for telemedicine follow-up management of patients after liver transplantation. Furthermore, the warning signs of complications (such as portal vein stenosis, bile leakage) were discovered earlier in the telemedicine group, and the patients received professional treatment in timely. There was no significant difference in the cumulative survival curves; however, there was a 2-year period of stability post–liver transplantation in the telemedicine follow-up group, and the cumulative survival rate was high (Figure 4). It might be associated with enhanced patient self-management and medication adherence through the telemedicine follow-up management system. Thus, the telemedicine follow-up management system could improve the patients’ health-related quality of life and facilitate achieving long-term outcomes in patients.

The influencing factors for long-term survival post–liver transplantation are numerous, complicated, and frequently associated with patient-specific risk factors (age, preoperative complications, disease severity, and donor conditions). Previously, being an older adult was considered to be a contraindication for being a donor due to the increased risk of poor graft function; however, subsequent studies [31] have indicated that liver grafts from donors ≥70 years old have outcomes similar to those of younger donors. Cumulative experiences with advanced age donors report excellent outcomes in this era of organ shortage and aging population. Moreover, the study of ex vivo machine perfusion of the liver is under investigation. Improvements in donor management, organ preservation, and mitigation of ischemia and reperfusion injury hold promise in allowing safe expansion of the donor pool and improvement of outcomes in the liver transplantation [32,33]. Our study indicated that telemedicine follow-up management system is closer to achieving textbook outcomes in liver transplantation. In the modern era of rapidly developing liver transplantation capabilities, we speculate that the textbook outcome in liver transplantation is cost-effective and useful as a composite metric to reflect the quality of perioperative care. Patients with challenging perioperative courses can be helped and might experience positive long-term outcomes. The telemedicine follow-up management in liver transplantation improved the quality of perioperative care and significantly reduced the readmission rate within 30 days after discharge; therefore, post–liver transplantation medical expenses were lower.

Patients in the telemedicine group in our study were satisfied with the telemedicine follow-up management system stating that it enhanced the sense of security and medication compliance after liver transplantation. It also saved costs and time in outpatient follow-up. Furthermore, the telemedicine follow-up management system saves time for transplant specialists, optimizes the allocation of medical resources, and promotes the early and rapid recovery of patients after liver transplantation. The telemedicine follow-up management system is highly beneficial to patients with poor recovery from severe complications post–liver transplantation by helping transplant specialists to closely monitor the patients’ condition after discharge and guide recovery.

Although no significant difference was detected in the diagnosis and treatment of postoperative complications between the telemedicine group and the usual care follow-up group, a large number of patients in the telemedicine group showed improved self-management and medication adherence. Additionally, early warning signs of complications were detected, and the patients received timely professional treatment. For example, a change was detected in the drainage fluid through remote video follow-up, the warning signs of portal vein stenosis were detected early in the telemedicine group, and the patients received professional treatment in a timely manner and improved the quality of life. Portal vein stenosis occurs in approximately 3% of liver transplantations but occurs in approximately 3.4% to 14% of split liver transplantations; early detection and treatment are essential for long-term graft survival [34,35]. Recently, some studies [36-38] highlighted the key role of interventional radiology in treating the stenosis safely and successfully with balloon angioplasty with stenting. In addition, patients could actively learn self-management and healthy exercise after liver transplantation. Robust physical activity after liver transplantation is a critical determinant of long-term health, similar that of pretransplant activity, for withstanding the immediate stress of transplantation [39].

Digital technology currently plays a major role in various fields. Digitization in medicine has been implemented for remote health monitoring, visual interactions between patient and doctor, and visual interactions between doctors from different hospitals and countries [40-42]. Increasing attention has been focused on the sustainability of health care systems; telemedicine allows health care providers to remotely diagnose and treat patients using telecommunications as either an alternative to or along with clinical visits [43,44]. Self-management support is one of the mechanisms by which telemedicine interventions have been proposed to facilitate the management of long-term conditions. In the last decade, telemedicine supported self-management of heart failure, asthma, chronic obstructive pulmonary disease, and cancer [45]. The most prominent examples within telehealth are related to pulmonary care: telemedicine with diagnosis at a distance based on spirometry tracing, teleconsultation, telemonitoring of biological signals, decision support systems, telecare, telerehabilitation, and second-opinion calls [16]. While telemedicine-mediated self-management was not consistently superior to that of usual care in several studies [45], none of the reviews reported negative effects, suggesting that it is a safe option for the delivery of self-management support. The key to optimizing the use of telemedicine is to correctly identify the ideal candidates, durations, and time points for a specific need [46].

In our study, the telemedicine follow-up management system was customized for patient post–liver transplantation, and the intervention administered for a short time after hospital discharge, which has not previously been done. We also emphasized the interaction between patient and transplant specialists, and rehabilitation guidance was provided according to the individual’s recovery early post–liver transplantation. The increasing number of patients requiring organ transplants, the complex landscape of liver transplantation, long distances, and poor road infrastructure between doctors and patients create barriers for the delivery of health care services, especially rural regions, some of which can be addressed by telemedicine. The telemedicine follow-up management system for liver transplantation promoted innovative treatment by accelerated exchange of patient data, and faster patient recovery is beneficial to both doctors and patients.

The development of telemedicine has some limitations. The most relevant factors in assessing the quality of telemedicine management are correct imaging, correct medical history, and the clinical skills of the physician. A 92% to 98％ diagnostic conformity was detected between telemedicine assessment and a face-to-face clinical assessment in a prospective pilot study [47]. Second, the misuse of personal data and information from patients’ medical documents is a significant issue. Unfair access to such personal and confidential information can be potentially dangerous [41]. Therefore, it is necessary to strengthen digital information security and formulate a relevant management system.

### Limitations

This study has several limitations. The follow-up intervention duration was only 2 weeks, and the number of patients was small. The generalizability of our results requires verification. Additionally, we could not determine whether telemedicine follow-up management differed between younger and older patients. However, our telemedicine follow-up management was customized in post–liver transplantation with emphasis on the interaction between patient and transplant specialists. In order to promote and apply to other fields, additional specific components of follow-up are essential. The telemedicine follow-up robot was inconvenient to carry; hence, a wireless network is required; however, some patients may not have access to a wireless network to be able to implement the program. Therefore, further improvement is required (for example, using 5G networks) to make it flexible and convenient.

### Conclusion

We demonstrated that rapid recovery and low readmission rate within 30 days after discharge were evident for telemedicine follow-up management of patients in the early stage, post–liver transplantation, which might be due to more efficient perioperative follow-up management. Furthermore, warning signs of complications were discovered early in the telemedicine group, and the patients received professional and timely treatment. The survival rate of patients in the telemedicine follow-up group was high in the first 2 years post–liver transplantation, which could be attributed to better patient self-management and medication adherence through the telemedicine follow-up management system. The telemedical management system is crucial in improving the patients’ health-related quality of life and achieving long-term outcomes in patients. Therefore, the intervention of the telemedicine follow-up management system is beneficial to achieving optimal clinical outcomes in liver transplantation.

## Abbreviations

COVID-19: coronavirus disease 2019
ECG: electrocardiography
MELD: Model for End-Stage Liver Disease

## References

[1] Starzl TE, Demetris AJ, Van Thiel D (1989). Liver transplantation (1). N Engl J Med.

[2] Adam R, Karam V, Delvart V, O'Grady J, Mirza D, Klempnauer J, Castaing D, Neuhaus P, Jamieson N, Salizzoni M, Pollard S, Lerut J, Paul A, Garcia-Valdecasas JC, Rodríguez Fernando San Juan, Burroughs A, All contributing centers (www.eltr.org), European Liver and Intestine Transplant Association (ELITA) (2012). Evolution of indications and results of liver transplantation in Europe. a report from the European Liver Transplant registry (ELTR). J Hepatol.

[3] Agopian V, Petrowsky H, Kaldas F, Zarrinpar A, Farmer D, Yersiz H, Holt Curtis, Harlander-Locke Michael, Hong Johnny C, Rana Abbas R, Venick Robert, McDiarmid Sue V, Goldstein Leonard I, Durazo Francisco, Saab Sammy, Han Steven, Xia Victor, Hiatt Jonathan R, Busuttil Ronald W (2013). The evolution of liver transplantation during 3 decades: analysis of 5347 consecutive liver transplants at a single center. Ann Surg.

[4] Kim WR, Lake JR, Smith JM, Schladt DP, Skeans MA, Harper AM, Wainright JL, Snyder JJ, Israni AK, Kasiske BL (2018). OPTN/SRTR 2016 annual data report: liver. Am J Transplant.

[5] Moris D, Tsilimigras DI, Ntanasis-Stathopoulos I, Beal EW, Felekouras E, Vernadakis S, Fung JJ, Pawlik TM (2017). Liver transplantation in patients with liver metastases from neuroendocrine tumors: a systematic review. Surgery.

[6] Moris D, Kostakis ID, Machairas N, Prodromidou A, Tsilimigras DI, Ravindra KV, Sudan DL, Knechtle SJ, Barbas AS (2019). Comparison between liver transplantation and resection for hilar cholangiocarcinoma: a systematic review and meta-analysis. PLoS One.

[7] Shah VH, Rao MK (2020). Changing landscape of solid organ transplantation for older adults: trends and post-transplant age-related outcomes. Curr Transplant Rep.

[8] Dutkowski P, Linecker M, DeOliveira ML, Müllhaupt Beat, Clavien P (2015). Challenges to liver transplantation and strategies to improve outcomes. Gastroenterology.

[9] Gao Q, Mulvihill MS, Scheuermann U, Davis RP, Yerxa J, Yerokun BA, Hartwig MG, Sudan DL, Knechtle SJ, Barbas AS (2019). Improvement in liver transplant outcomes from older donors: a US national analysis. Ann Surg.

[10] Taylor R, Allen E, Richards JA, Goh MA, Neuberger J, Collett D, Pettigrew GJ, Liver Advisory Group to NHS Blood and Transplant (2019). Survival advantage for patients accepting the offer of a circulatory death liver transplant. J Hepatol.

[11] Zheng Shu-Sen, Xu Xiao, Wu Jian, Chen Jun, Wang Wei-Lin, Zhang Min, Liang Ting-Bo, Wu Li-Ming (2008). Liver transplantation for hepatocellular carcinoma: Hangzhou experiences. Transplantation.

[12] Wang LY, Zheng SS (2018). Advances in predicting the prognosis of hepatocellular carcinoma recipients after liver transplantation. J Zhejiang Univ Sci B.

[13] Wang Fu-Sheng, Fan Jian-Gao, Zhang Zheng, Gao Bin, Wang Hong-Yang (2014). The global burden of liver disease: the major impact of China. Hepatology.

[14] Kolfschoten NE, Kievit J, Gooiker GA, van Leersum NJ, Snijders HS, Eddes EH, Tollenaar RAEM, Wouters MWJM, Marang-van de Mheen PJ (2013). Focusing on desired outcomes of care after colon cancer resections; hospital variations in 'textbook outcome'. Eur J Surg Oncol.

[15] Moris D, Shaw BI, Gloria J, Kesseli SJ, Samoylova ML, Schmitz R, Manook M, McElroy LM, Patel Y, Berg CL, Knechtle SJ, Sudan DL, Barbas AS (2020). Textbook outcomes in liver transplantation. World J Surg.

[16] Ambrosino N, Vitacca M, Dreher M, Isetta V, Montserrat JM, Tonia T, Turchetti G, Winck JC, Burgos F, Kampelmacher M, Vagheggini G, ERS Tele-Monitoring of Ventilator-Dependent Patients Task Force (2016). Tele-monitoring of ventilator-dependent patients: a European Respiratory Society statement. Eur Respir J.

[17] Daniel H, Sulmasy LS, HealthPublic Policy Committee of the American College of Physicians (2015). Policy recommendations to guide the use of telemedicine in primary care settings: an American College of Physicians position paper. Ann Intern Med.

[18] Cohen JM, Bunick CG, Perkins SH (2020). The new normal: an approach to optimizing and combining in-person and telemedicine visits to maximize patient care. J Am Acad Dermatol.

[19] Yen YH, Tsai YF, Su VY, Chan SY, Yu WR, Ho H, Hou CM, Chen CC, Woung LC, Huang SJ (2020). Use and cost-effectiveness of a telehealth service at a centralized COVID-19 quarantine center in Taiwan: cohort study. J Med Internet Res.

[20] Ambrosino N, Vagheggini G, Mazzoleni S, Vitacca M (2016). Telemedicine in chronic obstructive pulmonary disease. Breathe (Sheff).

[21] Culmer N, Smith T, Stager C, Wright A, Burgess K, Johns S, Watt M, Desch M (2020). Telemedical asthma education and health care outcomes for school-age children: a systematic review. J Allergy Clin Immunol Pract.

[22] Koehler F, Koehler K, Deckwart O, Prescher S, Wegscheider K, Kirwan B, Winkler S, Vettorazzi E, Bruch L, Oeff M, Zugck C, Doerr G, Naegele H, Störk Stefan, Butter C, Sechtem U, Angermann C, Gola G, Prondzinsky R, Edelmann F, Spethmann S, Schellong SM, Schulze PC, Bauersachs J, Wellge B, Schoebel C, Tajsic M, Dreger H, Anker SD, Stangl K (2018). Efficacy of telemedical interventional management in patients with heart failure (TIM-HF2): a randomised, controlled, parallel-group, unmasked trial. The Lancet.

[23] Penninga L, Lorentzen AK, Davis C (2020). A telemedicine case series for acute medical emergencies in Greenland: a model for austere environments. Telemed J E Health.

[24] Gregory ME, Sonesh SC, Hughes AM, Marttos A, Schulman CI, Salas E (2020). Using telemedicine in mass casualty disasters. Disaster Med Public Health Prep.

[25] Yardley L, Joseph J, Michie S, Weal M, Wills G, Little P (2010). Evaluation of a web-based intervention providing tailored advice for self-management of minor respiratory symptoms: exploratory randomized controlled trial. J Med Internet Res.

[26] Liu WT, Huang CD, Wang CH, Lee KY, Lin SM, Kuo HP (2011). A mobile telephone-based interactive self-care system improves asthma control. Eur Respir J.

[27] Burgos F, Disdier C, de Santamaria EL, Galdiz B, Roger N, Rivera ML, Hervàs R, Durán-Tauleria E, Garcia-Aymerich J, Roca J, e-Spir@p Group (2012). Telemedicine enhances quality of forced spirometry in primary care. Eur Respir J.

[28] Xu Xiao, Chen Jun, Wei Qiang, Liu Zhi-Kun, Yang Zhe, Zhang Ming, Wang Guo-Ying, Gao Jie, Yang Zhao-Xu, Guo Wen-Yuan, Xing Tong-Hai, Shao Zhou, Xie Qin-Fen, Zheng Shu-Sen (2019). Clinical practice guidelines on liver transplantation for hepatocellular carcinoma in China (2018 edition). Hepatobiliary Pancreat Dis Int.

[29] Li Y, Sun H, Yan X, Wang S, Dong D, Liu X, Wang B, Su M, Lv Y (2020). Magnetic compression anastomosis for the treatment of benign biliary strictures: a clinical study from China. Surg Endosc.

[30] Zhao G, Yan X, Ma L, Liu W, Zhang J, Guo H, Liu Y, Lv Y (2019). Biomechanical and performance evaluation of magnetic elliptical-ring compressive anastomoses. J Surg Res.

[31] Emre S, Schwartz ME, Altaca G, Sethi P, Fiel MI, Guy SR, Kelly DM, Sebastian A, Fisher A, Eickmeyer D, Sheiner PA, Miller CM (1996). Safe use of hepatic allografts from donors older than 70 years. Transplantation.

[32] Boteon YL, Afford SC (2019). Machine perfusion of the liver: Which is the best technique to mitigate ischaemia-reperfusion injury?. World J Transplant.

[33] Rijkse E, IJzermans JN, Minnee RC (2020). Machine perfusion in abdominal organ transplantation: Current use in the Netherlands. World J Transplant.

[34] Orons PD, Zajko AB, Bron KM, Trecha GT, Selby RR, Fung JJ (1995). Hepatic artery angioplasty after liver transplantation: experience in 21 allografts. J Vasc Interv Radiol.

[35] Cheng YF, Ou HY, Tsang LL, Yu CY, Huang TL, Chen TY, Concejero A, Wang CC, Wang SH, Lin TS, Liu YW, Yang CH, Yong CC, Chiu KW, Jawan B, Eng HL, Chen CL (2010). Vascular stents in the management of portal venous complications in living donor liver transplantation. Am J Transplant.

[36] Yabuta M, Shibata T, Shibata T, Shinozuka K, Isoda H, Okamoto S, Uemoto S, Togashi K (2014). Long-term outcome of percutaneous transhepatic balloon angioplasty for portal vein stenosis after pediatric living donor liver transplantation: a single institute's experience. J Vasc Interv Radiol.

[37] Thornburg B, Katariya N, Riaz A, Desai K, Hickey R, Lewandowski R, Salem R (2017). Interventional radiology in the management of the liver transplant patient. Liver Transpl.

[38] Prasad R, Yadav RR, Israrahmed A, Mittal SR (2020). Endovascular management in post liver transplant recipients with venous anastomotic site stenosis and an associated iatrogenic arterio-portal fistula: case series and review of literature. J Clin Diagn Res.

[39] Dunn MA, Rogal SS, Duarte-Rojo A, Lai JC (2020). Physical function, physical activity, and quality of life after liver transplantation. Liver Transpl.

[40] Raeesi Vanani I, Amirhosseini M, Chakraborty C, Banerjee A, Kolekar M, Garg L, Chakraborty B (2020). IoT-based diseases prediction and diagnosis system for healthcare. Internet of Things for Healthcare Technologies Studies in Big Data, vol 73.

[41] Mirskikh I, Mingaleva Z, Kuranov V, Matseeva S, Antipova T (2020). Digitization of medicine in Russia: mainstream development and potential. Integrated Science in Digital Age Lecture Notes in Networks and Systems, vol 136.

[42] Ye Junna, Zuo Yanhai, Xie Ting, Wu Minjie, Ni Pengwen, Kang Yutian, Yu Xiaoping, Sun Xiaofang, Huang Yao, Lu Shuliang (2016). A telemedicine wound care model using 4G with smart phones or smart glasses: a pilot study. Medicine (Baltimore).

[43] Cowie MR, Bax J, Bruining N, Cleland JGF, Koehler F, Malik M, Pinto F, van der Velde E, Vardas P (2016). e-Health: a position statement of the European Society of Cardiology. Eur Heart J.

[44] Marx G, Beckers R, Brokmann JC, Deisz R, Pape HC (2015). Tele-cooperation for innovative care using the example of the University Hospital Aachen. Telematics in intensive care medicine, emergency medicine, and telemedical intersectoral rehabilitation planning in geriatric trauma. Bundesgesundheitsblatt Gesundheitsforschung Gesundheitsschutz.

[45] Hanlon P, Daines L, Campbell C, McKinstry B, Weller D, Pinnock H (2017). Telehealth interventions to support self-management of long-term conditions: a systematic metareview of diabetes, heart failure, asthma, chronic obstructive pulmonary disease, and cancer. J Med Internet Res.

[46] Vitacca M, Montini A, Comini L (2018). How will telemedicine change clinical practice in chronic obstructive pulmonary disease?. Ther Adv Respir Dis.

[47] Villa L, Matz O, Olaciregui Dague K, Kluwig D, Rossaint R, Brokmann JC (2020). The assessment of dermatological emergencies in the emergency department via telemedicine is safe: a prospective pilot study. Intern Emerg Med.

